# Compensatory Interactions between Sir3p and the Nucleosomal LRS Surface Imply Their Direct Interaction

**DOI:** 10.1371/journal.pgen.1000301

**Published:** 2008-12-12

**Authors:** Anne Norris, Mario A. Bianchet, Jef D. Boeke

**Affiliations:** 1 Department of Molecular Biology and Genetics, Johns Hopkins University School of Medicine, Baltimore, Maryland, United States of America; 2 The High Throughput Biology Center, Johns Hopkins University School of Medicine, Baltimore, Maryland, United States of America; 3 Department of Biophysics, Johns Hopkins University School of Medicine, Baltimore, Maryland, United States of America; Yale University, United States of America

## Abstract

The previously identified LRS (Loss of rDNA Silencing) domain of the nucleosome is critically important for silencing at both ribosomal DNA and telomeres. To understand the function of the LRS surface in silencing, we performed an EMS mutagenesis screen to identify suppressors of the H3 A75V LRS allele. We identified dominant and recessive mutations in histones H3, H4, and dominant mutations in the BAH (Bromo Adjacent Homology) domain of *SIR3*. We further characterized a surface of Sir3p critical for silencing via the LRS surface. We found that all alleles of the *SIR3* BAH domain were able to 1) generally suppress the loss of telomeric silencing of LRS alleles, but 2) could not suppress SIN (Swi/Snf Independent) alleles or 3) could not suppress the telomeric silencing defect of H4 tail alleles. Moreover, we noticed a complementary trend in the electrostatic changes resulting from most of the histone mutations that gain or lose silencing and the suppressor alleles isolated in *SIR3*, and the genes for histones H3 and H4. Mutations in H3 and H4 genes that lose silencing tend to make the LRS surface more electronegative, whereas mutations that increase silencing make it less electronegative. Conversely, suppressors of LRS alleles in either *SIR3*, histone H3, or H4 also tend to make their respective surfaces less electronegative. Our results provide genetic evidence for recent data suggesting that the Sir3p BAH domain directly binds the LRS domain. Based on these findings, we propose an electrostatic model for how an extensive surface on the Sir3p BAH domain may regulate docking onto the LRS surface.

## Author Summary

The chromatin in which eukaryotic DNA is wrapped is organized in nucleosome units, consisting of eight core histone molecules. We study gene silencing, thought to be associated with chromatin compaction in *Saccharomyces cerevisiae*, which has most of its genome in the form of open chromatin, i.e., the genes are expressed. There are three distinct classes of silent genomic loci associated with such a compact structure; when genes are inserted at these loci, they are subject to silencing, regardless of DNA sequence. Yeast cells distinguish between silent and expressed loci using silencing complexes, concentrated at silent loci, as well as establishing different modification states of silent and expressed chromatin. One of these silencing proteins, Sir3p, is crucial for silencing transcription. Using suppressor genetics, we identified a genetic interaction between a specific surface patch on the nucleosome face (the LRS surface) and Sir3p; the complementary charges of the respective surfaces determine the strength of this key interaction. Genetically guided molecular docking experiments identified electrostatic and steric complementarity between these two surfaces at the molecular level that could help explain both the genetics and the impact of the critical nucleosomal modifications that control silent chromatin formation.

## Introduction

Previous work identified a nucleosome surface named the LRS domain critically important for silencing at all classically defined silent loci in *Saccharomyces cerevisiae* [1],[2]. The relevant residues are located at Super Helical Location (SHL)+/−2.5 (equivalent to 4 o'clock on the nucleosome face, with 12 o'clock being the dyad axis) [3]. These residues surround histone H3 K79, the site of Dot1p methylation that regulates silencing [4],[5]. Bulk nucleosomes are 90% methylated at H3K79 and this modification is widely distributed across the euchromatic yeast genome but markedly depleted at heterochromatic mating-type, ribosomal DNA, and telomeric loci. These results suggest that this residue is important for defining euchromatin, and imply that the absence of such methylation defines a silent chromatin ground state [4]–[7]. Most of the genome of *S. cerevisiae* is in the active state, as genes are densely spaced and most of the genes are expressed under standard laboratory growth conditions. There are three regions of silent chromatin, the silent mating type cassettes or *HM* loci, the telomeres, and the rDNA repeats (reviewed in [8]). Three models have been put forth to explain the role of the LRS domain in silencing: (i) these residues are simply directly required for K79 methylation/Dot1p recognition, (ii) the LRS surface could represent a direct nucleosome-nucleosome interaction surface important for tight packing of nucleosomes and silencing (iii), the surface may represent a site of interaction between the nucleosome and a silencing protein(s) [1]. While there is evidence against the first two models, until recently data supporting the third hypothesis, which we favor, were lacking. The evidence against the first two hypotheses is as follows: i) Most LRS alleles are competent for Dot1p methylation [9]; (ii) despite the fact that many of LRS residues are important for DNA binding, we showed that the LRS surface is not required structurally for condensation of oligonucleosome arrays *in vitro* [9]. Moreover, one model for a condensed chromatin fiber, based on the tetranucleosome structure, suggests that LRS surfaces are solvent-exposed and not involved in intimate nucleosome-nucleosome interactions [10]. (iii) Data presented here and elsewhere [11],[12] provide evidence that the LRS surface directly binds the Sir3p BAH domain, and this binding is important for silencing at the telomeres.

In the current model for telomeric silencing, Rap1 and Ku proteins bind directly to telomere chromatin, followed by recruitment of the Sir complex (Sir2/3/4p). Iterative cycles of deacetylation of histone tails, and specifically H4 K16, by Sir2p create high-affinity sites for the Sir 2/3/4 complex in adjacent nucleosomes, allowing spreading of silent chromatin [13]–[17]. The encroachment of silent chromatin from silent loci into neighboring euchromatin is prevented by several redundant mechanisms. The modification of residues of H3 K4 and H3 K79 and H4 K16 are important for telomeric silencing. Methylation of both H3 K4 and K79 and acetylation of H4 K16 in active chromatin are needed to restrict Sir3p to silent chromatin [4]–[6], [18]–[20]. Recent work has given insight into how these modifications restrict silent chromatin. These data suggest that Sir3p and Dot1p, the methyl transferase for H3 K79, compete for binding of overlapping nucleosome surfaces consisting of the short basic patch of the H4 tail surrounding K16 as well as the region of the nucleosome core surrounding H3 K79 (the LRS surface), for overall chromatin binding affinity. Importantly, the Sir3p *in vitro* interaction is inhibited by both H4 K16 acetylation and H3 K79 methylation, whereas Dot1p binds irrespective of K16 acetylation status [11],[21],[22]. This scheme sets up a two fold level of regulation, the first level of competition between for the Sas2p, the H4 K16 acetyl transferase and Sir2p, the deacetylase, for the acetylation status of H4 K16 affects the second level of competition between Dot1p and Sir3p for the nucleosome surface. The region of Sir3p that is responsible for binding the nucleosome, and whether the interaction is direct remains unclear. Yeast two hybrid, *in vitro* and *in vivo* studies implicated the Sir3p C-terminus in interaction with Sir4p, Rap1p, itself, H3 and H4 tails, and the region around H3 K79 [14], [23]–[27]. However, point mutations in the N-terminal BAH region can weaken or abolish silencing, [28] and recent studies provide evidence that the N-terminal BAH domain of Sir3p is important for nucleosome binding [11],[12]. In this paper, we provide a genetic confirmation of the Sir3 BAH-LRS surface interaction that suggests it represents an extensive direct interaction surface dominated by the overall complementary charge states of the two surfaces. Based on these genetic data we propose a model of how the Sir3p BAH domain docks onto the nucleosome face.

## Results

### Nucleosome Electrostatics

**Figure 1:**
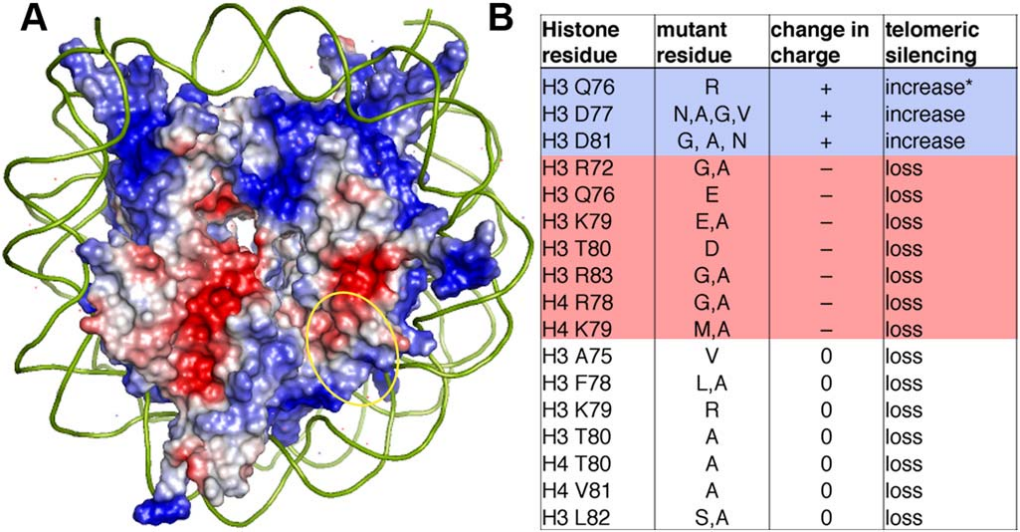
LRS residues affect the electrostatics of the nucleosome. (A) Qualitative vacuum electrostatic representation of the nucleosome 1ID3 [29] rendered using PyMOL [62]. Red is electronegative and blue is electropositive. (B) A table of residues found in several studies showing that an increase in positive charge in LRS residues leads to an increase in silencing, while a decrease in charge leads to loss of telomeric silencing. * The mutation Q76 to R was found to have an increase in spreading of silent chromatin [61]. Data was compiled from the following references [1],[2],[9],[31],[32],[61].

On the disk face of the nucleosome, there are two electronegative (red) surfaces, one of which partially overlaps the LRS surface ([29], K. Luger, personal communication, Figure 1) The other, stronger of the two, is at the H2A/H2B interface and has been shown to bind in trans to the H4 tail and make an important crystal contact in the *Xenopus* nucleosome structure [3]. The LRS region of the nucleosome consists of H3 residues 72–83 and H4 residues 78–81; data from both directed and random mutagenesis studies reveal an overwhelming trend in that all mutations in the LRS region that lose silencing either remove positive charge from the LRS surface or do not detectably affect the LRS surface potential. Importantly, no LRS allele was found to alter an acidic residue (Figure 1). In fact, mutations that increase the positive charge on this surface such as mutations in H3 D77(G,V,N), and H3 D81(G,N) actually increase telomeric silencing [1], [2], [9], [30]–[32]. The only exception to this trend is H3 E73, which when mutated to any residue regardless of charge loses telomeric silencing, but increases rDNA silencing. Based on these observations, we hypothesized that the LRS surface is a binding site for a trans-acting silencing factor, and that this interaction might be electrostatic in nature.

### Genetic Screen To Identify Suppressors of the LRS Surface

To investigate the function of the LRS surface we undertook an EMS screen for suppressors of the loss of telomeric silencing phenotype of one of the non-charge-altering LRS mutants, H3 A75V. H3 A75V was chosen because it showed a severe loss of both telomeric and rDNA silencing. We designed a telomeric silencing reporter strain lacking endogenous copies of all histone H3 and H4 genes, with a wild type H4 (*HHF2*) gene and H3-A75V (*HHT2-A75*) expressed from a centromeric plasmid. The strain contained a two-tiered reporter system described by Smith et al [31],[33]. In this strain, *ADE2* is located adjacent to the truncated right telomere of chromosome *V* (V-R) and *URA3* next to the truncated left telomere of chromosome *VII* (VII-L). The *ADE2* reporter gene displays a range of colors from dark red indicating most intense silencing to white indicating complete loss of silencing. Colonies of wild-type yeast display an intermediate phenotype, with sectors of red and white, reflecting epigenetic switching between states [33]. Similarly, silencing of the telomeric *URA3* reporter can be monitored quantitatively by growth on synthetic medium lacking uracil, or on counter-selective medium containing 5-FOA (5-fluoroorotic acid). The *URA3* coactivator, *PPR1*, was also deleted, rendering the reporter strain completely 5-FOA sensitive in the presence of histone H3 LRS mutant H3-A75V, but 5-FOA resistant with wild-type histones. Suppressors of H3-A75V were isolated by selection on 5-FOA. We mutagenized approximately 10^9^ cells, yielding 44 independent plasmid-borne mutations and 40 independent genomic mutations (summarized in Table 1).

**Table 1:** Summary of EMS mutagenesis.

Gene	Mutations	Dominant or Recessive
*SIR3*	D205N(38)	Dominant
	L79I (2)	Dominant
*HHT2* (H3)	R39K (8)	Recessive
	D77N (31)	Dominant
	D77G (2)	Dominant
*HHF2* (H4)	A15V (1)	Recessive
	H75Y (2)	Recessive

The gene mutated is indicated in the left column. The mutations and number of independent isolations of a mutation are indicated in parentheses.

### Histone Suppressor Mutations

**Figure 2:**
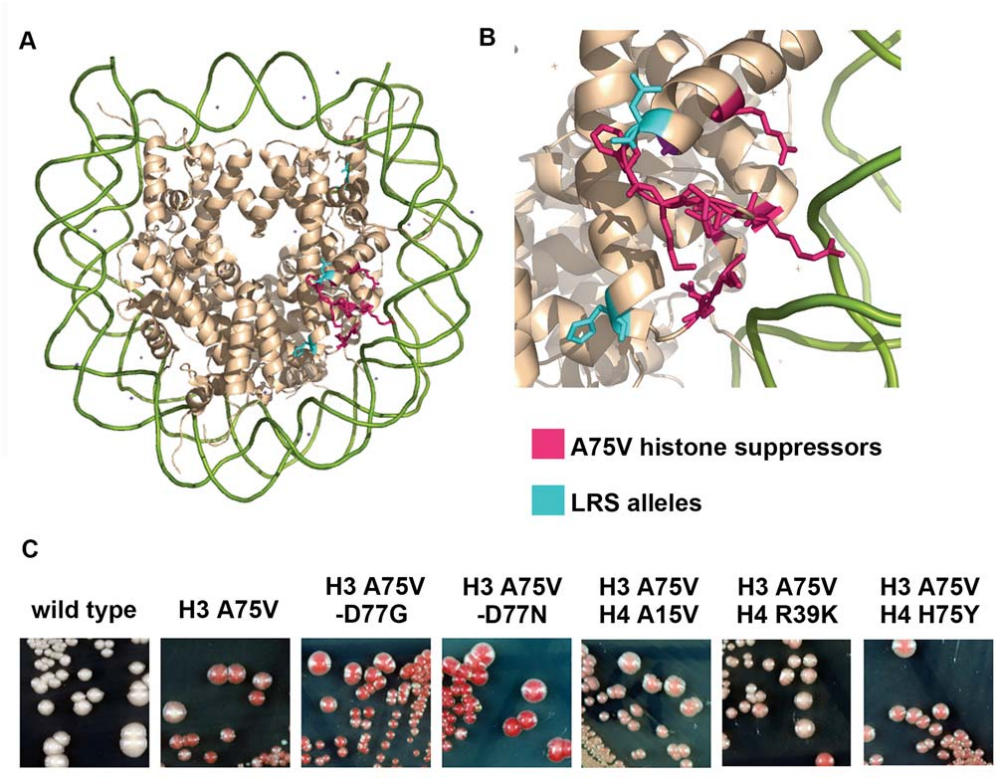
Histone suppressors of H3 A75V. (A) Disc face representation of the nucleosome 1ID3 [29] rendered using PyMOL [62]. (B) Zoom view of the LRS surface of the disc face. The DNA is represented in green, the histones are represented in wheat, the LRS residues and their side chains are highlighted in magenta, and the suppressor alleles are highlighted in cyan. (C) Telomeric *ADE2* silencing with wild-type, H3 A75V and H3 A75V with histone suppressors.

**Figure 3:**
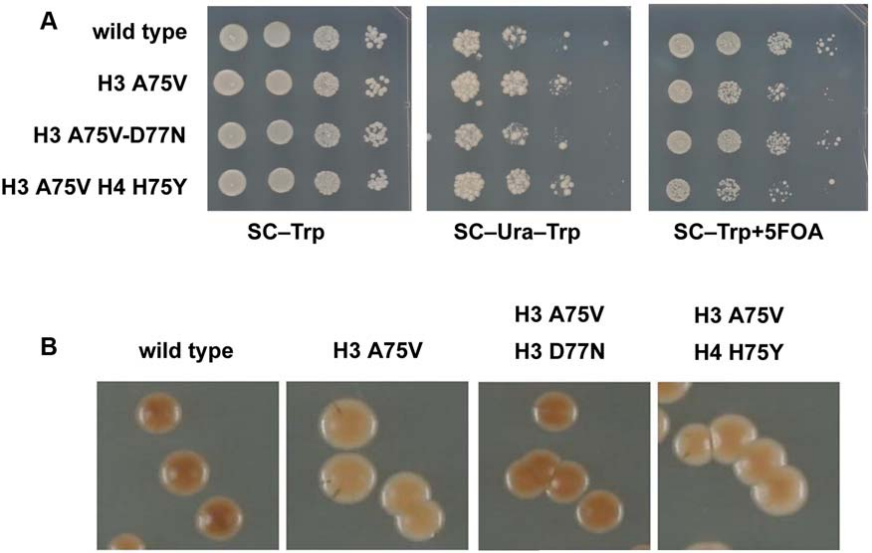
Effect of histone mutations on rDNA silencing. H3 D77N but not H4 H75Y suppresses the loss of rDNA silencing phenotype of H3 A75V. JPY12 yeast strain with the histone alleles on a plasmid were plated as indicated. (A) SC−Trp for growth control and SC−URA or +FOA to measure rDNA silencing. (B) MLA plates assaying for silencing of the *MET15* reporter inserted into the *NTS2* of the rDNA. Dark color indicates increased silencing.

Based on the electrostatic properties of the LRS surface, we predicted that histone mutations that decreased the negative charge of the LRS surface might restore telomeric silencing. The only copies of histone H3 and H4 genes in the reporter strain were supplied on a centromeric plasmid, thus potential suppressor mutations in H3 and H4 were easily identified by a plasmid shuffling strategy. 44 of the isolates displayed a plasmid-dependent phenotype. We recovered the original suppressor-containing plasmids and identified the following inter- and intragenic histone suppressor mutations: H4 A15V, H75Y, R39K, and H3 D77N, and D77G (Table 1 and Figure 2). Remarkably, an independent screen for H3 and H4 mutations that increased telomeric silencing in a *cac1Δ* background also revealed these same H3 and H4 alleles [31]. The most potent of these mutants, and the only dominant alleles among them, H3 D77N and D77G, are located on the LRS surface and are predicted to decrease its negative charge. Additionally D77N, but not H75Y suppresses the loss of rDNA silencing phenotype of H3 A75V (Figure 3).

### Chromosomal Suppressor Mutations

**Figure 4:**
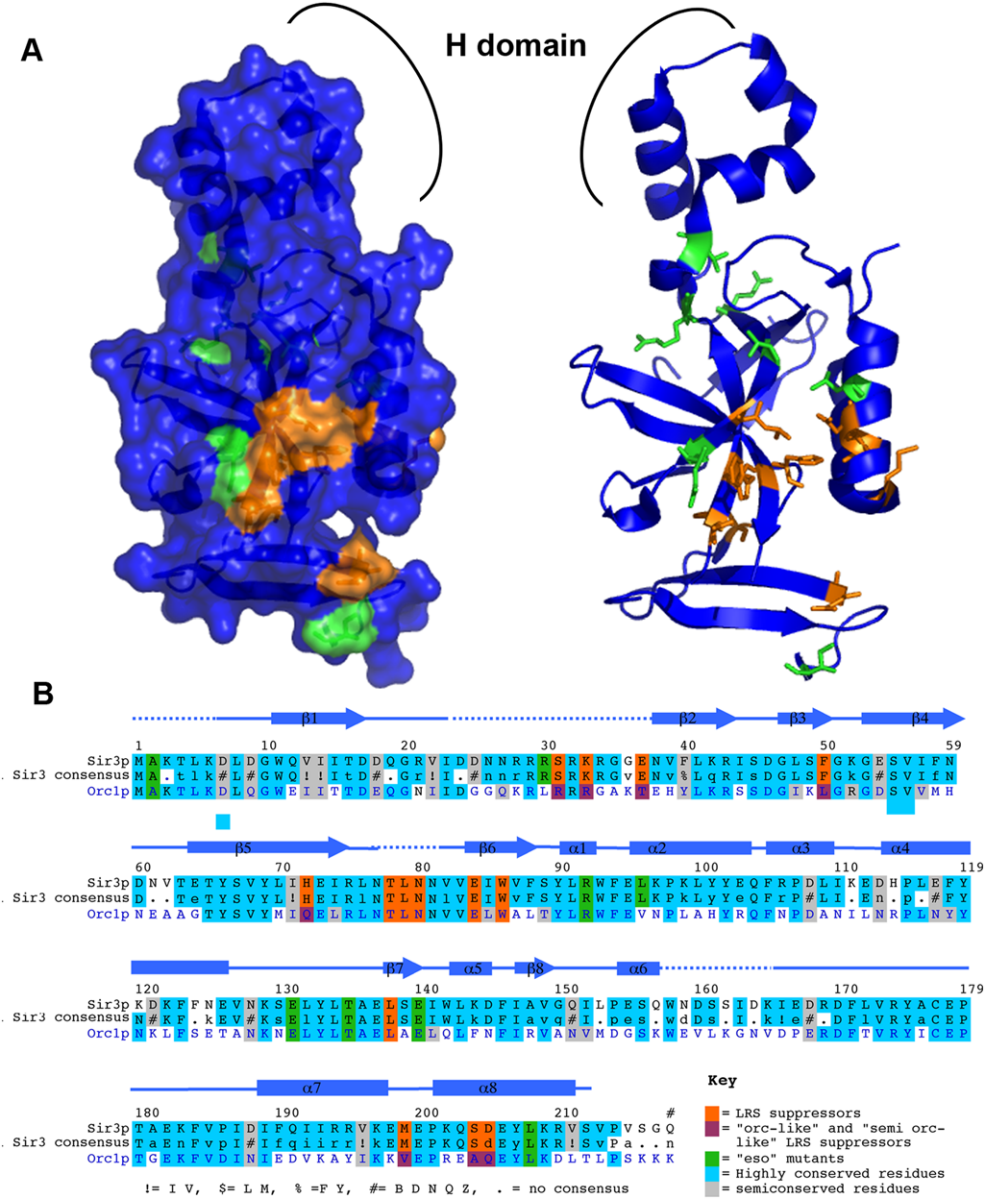
*SIR3* suppressor mutants define a surface of Sir3p. (A) Mapping *SIR3* mutants onto the crystal structure of the Sir3p BAH domain 2fl7 [35]. Mutants identified in both the EMS screen and the PCR mutagenesis of the BAH domain are represented in orange. *SIR3 eso* mutants are represented in green [28]. (B) An alignment of Sir3p and Orc1p BAH domains with H3 A75V and *eso* mutants highlighted. Many of the mutants are disordered in the crystal structure, the approximate locations of missing sections, and are represented by dashed lines. Residues important for suppression of H3 A75V are highlighted in orange. H3 A75V suppressor mutants that introduce an “Orc1p like” residue are highlighted in magenta; *eso* mutants are highlighted in green. Highly conserved and semiconserved residues are highlighted in light blue and gray, respectively.

The remaining 40 dominant suppressor mutants retained their H3-A75V suppressor phenotype after retransformation with a “clean” histone plasmid. These mutations were mapped using a new TAG array based meiotic mapping procedure, TaGAM (Norris and Boeke, unpublished data) followed by DNA sequencing. All of the non-histone suppressors mapped to *SIR3*. Of these *SIR3* suppressor mutants, 38 carried the *SIR3-D205N* allele and 2 carried the novel allele *SIR3-L79I*, both located in the N-terminal BAH domain of *SIR3* (Figure 4). Interestingly, the *SIR3-D205N* mutation was previously isolated as a suppressor of the mating defect of a histone H4 K16Q tail mutation [27] and also as a suppressor of a *rap1-17* allele incapable of recruiting Sir3p to the telomere [24], consistent with a general gain of function or “super-*SIR3*” phenotype for this allele. Additionally, a bacterially expressed Sir3p-D205N BAH domain reportedly binds to nucleosomes and naked DNA in vitro with a higher affinity than the wild type [34]. The L79I substitution lies in a hydrophobic loop between β5 and β6; this protein segment is disordered in the existing crystal structures of the Sir3p BAH domain [34],[35] (see Figure 4).

### *SIR3-D205N* and *L79I* Specifically Suppress LRS Surface Mutations

**Figure 5:**
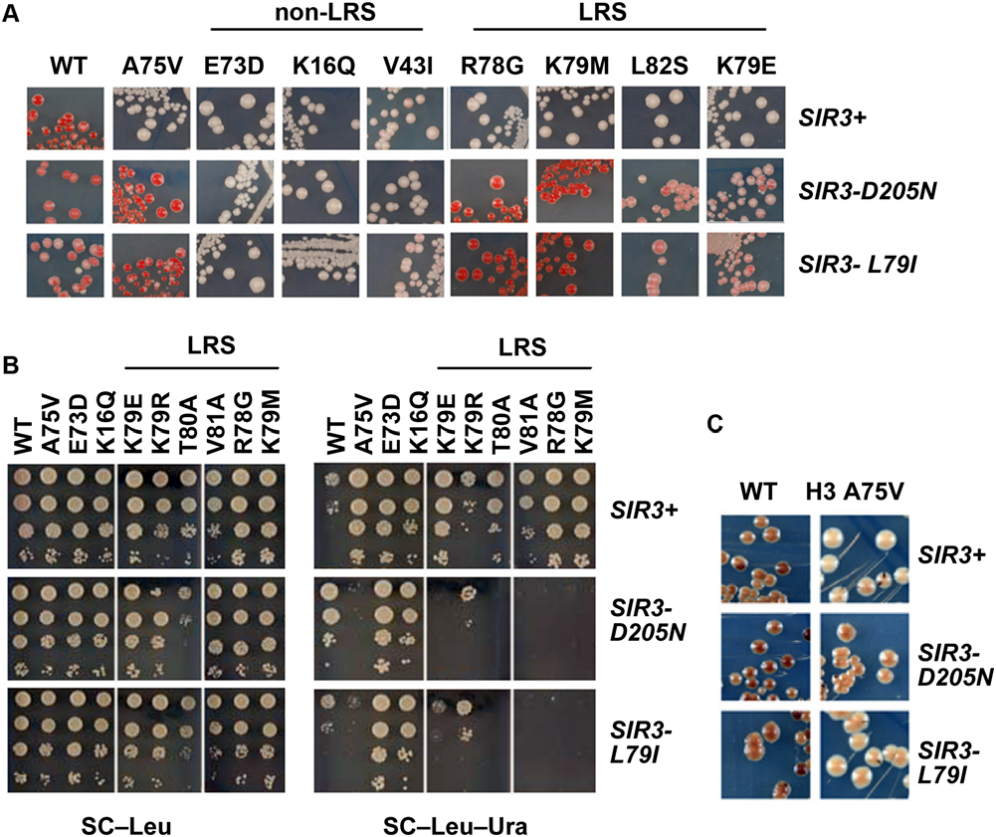
SIR3 D205N and L79I specifically suppress the LRS surface telomeric silencing defect. *SIR3* alleles L79I and D205N were assayed for their ability to suppress the loss of telomeric silencing of LRS alleles as well as other Histone alleles not on the LRS surface that lose telomeric silencing. (A) The *ADE2* reporter gene was assayed on SC−Trp. A pink color indicates silencing, whereas a lighter color indicates a loss of silencing. (B) The *URA3* gene was assayed for growth by serial dilution on SC−Ura. Increased growth indicates a loss of silencing, whereas decreased growth indicates an increase in silencing. (C) JPY12 yeast strain with the *SIR3* alleles on a plasmid were plated on MLA plates assaying for silencing of the *MET15* reporter in the rDNA.

It was remarkable that, given the whole genome as the target, only mutations in histones and *SIR3* suppressed H3 A75V loss of telomeric silencing. This result strongly supports a model in which Sir3p interacted directly with the LRS domain at the telomeres but did not rule out an indirect effect. To test the hypothesis that the Sir3p BAH domain had a specific interaction with the LRS surface of the nucleosome, we tested for the ability of *SIR3-D205N* and *SIR3-L79I* to suppress other histone mutations that lose telomeric silencing. Three different nucleosome regions are important for telomeric silencing, the H3 and H4 N terminal tails, the SIN (Swi/Snf Independent) surface and the LRS surface [1],[2],[9],[30],[31]. The SIN surface is located at the dyad axis, where the DNA enters and exits the nucleosome. The *sin* mutant alleles partially bypass the need for the SWI/SNF chromatin remodeling complex, are defective in nucleosome array formation, and defective for telomeric silencing. Additionally, these alleles also display defects in localization of Sir2p and Sir4p to the telomeres [9],[36]. If there were a specific interaction between the LRS surface and the Sir3p BAH domain we would expect the *SIR3* alleles to suppress the LRS mutants' defect in telomeric silencing but not the defects of SIN or other alleles. Indeed both *SIR3-D205N* and *SIR3- L79I* suppress the telomeric silencing phenotype of LRS alleles but not SIN allele H4 H4-V43I or other non LRS Histone alleles such as H3 E73D or H4 K16Q (Figure 5A and B). It should be noted however, that both the *SIR3-D205N* and *SIR3-L79I* do suppress the mating defects of both H3 E73D and H4 K16Q (data not shown), suggesting that Sir3p may bind somewhat differently at the two silent loci. Alternatively, these *SIR3* alleles may impart a small amount of restoration of silencing to *HM* loci sufficient to restore mating, but they do not have much effect on telomeric silencing. We found that these *SIR3* mutations do not restore rDNA silencing in H3 A75V (Figure 5C), as expected, given that Sir3p is not a part of the silencing complex important for rDNA [37],[38].

### *SIR3* Alleles Restore Sir3p Binding to Telomeric Chromatin in LRS Mutants

**Figure 6:**
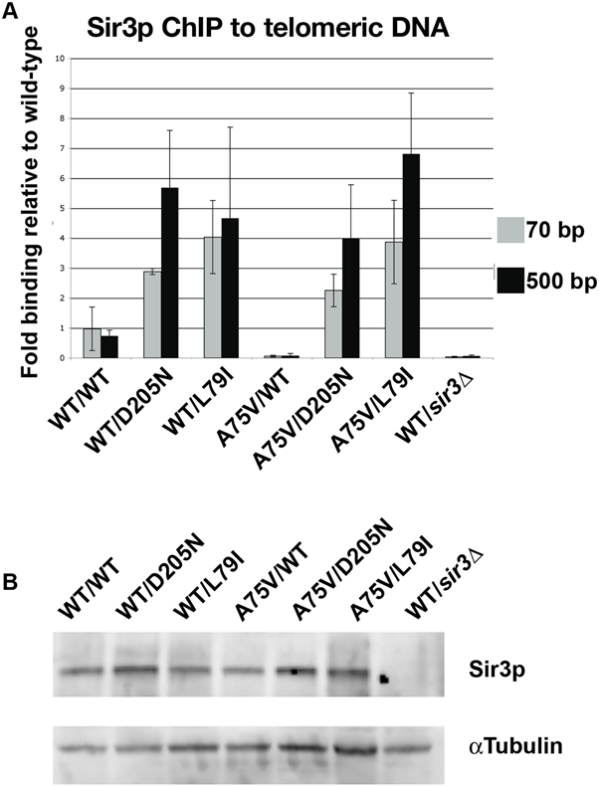
*SIR3* alleles restore Sir3p binding to telomeric DNA. (A) ChIP of Sir3p to telomeric DNA. The histone allele is indicated, followed by the *SIR3* allele on a *CEN* plasmid for each strain tested. Wt is wild-type, A75V is *hht2-A7V5*, D205N is *sir3-D205N*, and L79I is *sir3–L79I*. Wild type histones with PRS415 vector and endogenous *SIR3* deleted served as a control. All values were normalized to input DNA and the PHO5 locus and wild type H3/SIR3+. Primers specific to subtelomeric sequences starting at 70-bp, and 500-bp, regions from the C1-3A repeats at the right end of Chromosome VI (Chr. VI-R). (B) Steady state Sir3p levels in strains used for ChIP analysis show that increased ChIP to telomeric DNA is not a consequence of over expression of Sir3p. Western blot of log phase cells expressing *SIR3* alleles and LRS alleles probed with antibodies to Sir3p and tubulin as a loading control. Wt is wild-type, and A75V is *hht2-A75V*. Wild type histones with PRS415 vector and endogenous *SIR3* deleted served as a control. Wt is wild-type, A75V is *hht2-A7V5*, D205N is *sir3-D205N*, and L79I is *sir3–L79I*.

The mechanism of restoration of silencing by *SIR3* in the H3 A75V strain could be due to restoration of binding of Sir3p to telomeric DNA or to some indirect effect on telomeric silencing. To provide further evidence against an indirect mechanism we localized Sir3p using chromatin immunoprecipitation (ChIP) to telomeric DNA in wild-type or H3 A75V strains with the *SIR3* alleles D205N and L79I. We found that indeed the H3 A75V mutant blocks wild-type Sir3p from binding to telomeric DNA, consistent with similar effects on Sir2p and Sir4p observed previously [9]. As predicted, Sir3p telomere localization was restored in both *hht2-A75V-SIR3-D205N* and *hht2-A75V-SIR3-L79I* strains (Figure 6A). This restoration of Sir3p binding is not a result of increased steady state levels of Sir3p (Figure 6B). Interestingly, Sir3-D205Np and Sir3-L79Ip also bind wild-type telomeric nucleosomes more efficiently than does wild-type Sir3p, suggesting a gain of function in these mutant proteins. The efficiency of Sir3p telomeric localization does not entirely parallel the telomeric silencing phenotype as assayed by the *URA3* and *ADE2* reporters. Paradoxically, both *SIR3-D205N* and -*L79I* alleles show slight decreases in silencing with wild-type histones as compared to wild-type Sir3p with wild-type histones (Figure 5A and 5B). Clearly, the telomeric silencing phenotype is determined by more than simply the quantity or extent of Sir3p bound. This may explain why we failed to observe any suppression of H3 A75V and other LRS alleles by simple over-expression of wild-type *SIR3* (data not shown).

### An Extended Surface of Sir3p Critical for Telomeric Silencing

To further characterize the surface of Sir3p important for suppression of the LRS surface, we selectively mutagenized the BAH domain of *SIR3* and selected for additional suppressors of H3 A75V or H4 R78G. We isolated many additional suppressor substitution mutants (Table 2, Figure 4). All of these mutants clustered on one face of the Sir3p BAH domain surface and are in residues that are 100% conserved among *S. paradoxus*, *S. mikatae*, *S. bayanus*, *S. kudriavzevii*, and *S. castelli*, except for *S. castelli* – the most distantly related species – that differed only in having a glutamyl residue at position 205 as opposed to the aspartyl residue found in all other species examined (Figure 4B). These suppressor residues are also surrounded by the previously identified *eso* (enhancer of *sir1* mating defect) mutants, suggesting that this is indeed an important functional surface of Sir3p. There are three notable structural features of the Sir3 BAH domain affected by these mutants were isolated: (i) there is a highly charged region between amino acids 24 and 34 that is disordered in the crystal structure in which both loss of function and gain of function (*ESO* or enhancer of Sir One) alleles of *SIR3* have been isolated (current study, [24],[28] and Danesh Moazed, pers. comm.); (ii) There is also an extended hydrophobic loop between residues 77 and 83, most of which is disordered in the crystal structure; (iii) Finally the helix α8 and the twisted beta sheet (see Figure 4B) make up a contiguous surface and is where the majority of mutants were isolated. The *SIR3* LRS suppressors isolated here follow the general electrostatic trend in that many (69%) of the mutations increase the positive charge of the BAH domain and none of them decrease it. We also tested the allele specificity of these *SIR3* A75V suppressor alleles; all additional suppressor alleles tested had the same specificity for the LRS surface as both *SIR3-D205N* and *SIR3-L79I* (data not shown).

**Table 2:** *SIR3* BAH mutants.

Sir3p BAH Residue	Mutant Residue	Change in Charge*	In Crystal Structure	Corresponding Orc1p BAH Residue	Previous Report(s)
K33	R	+	No	**R**	
E37	G	+	Yes	T	
E84	A	+	Yes	E	
W86	R	+	Yes	W	W86R [27]
D205	N,G,V,I,S	+,+,+,+,+	Yes	Q	D205N [24],[27]
H72	Q,R	0, +	Yes	**Q**	
S31	L,T	0	No	R	S31L [24]
F50	L	0	Yes	**L**	
T78	A	0	No	T	
L79	I	0	No	L	
N80	S	0	No	N	
L138	P,Q	0	Yes	L	
M199	V	0	Yes	**V**	
S204	F	0	Yes	A	

Bold residues indicate that the mutant residue is the same as the corresponding Orc1p residue. An underlined residue indicates mutant residues that are not conserved between ORC1p and Sir3p suppressor mutants.

*: +, mutant is more positively charged than wild type; 0, no change in charge; −, mutant is more negatively charged than the wild type.

### Sir3 Mutations Are “Orc1p-Like”

Both Sir3p and the essential replication origin binding protein Orc1p contain BAH domains [39]. The two BAH domains are 55% identical and 76% similar [34]. Many of the suppressor alleles in *SIR3* are in residues that are not conserved between the Orc1p and Sir3p BAH domains. Of 21different independently isolated suppressor mutations, 14 fall in nonconserved residues. Remarkably, 4 of these are changes in Sir3p that introduce the corresponding Orc1p residue. Additionally, the frequently isolated D205N mutant changes to (N) asparagine at a position where Orc1p has the closely related (Q) glutamine. These “Orc-like” mutations do not lie in the H subdomain of the BAH domain, the Orc1p domain responsible for Sir1p binding. [40] (Figure 4). This result argues against the possibility that the gain of function nature of these *SIR3* alleles is mediated by an Orc-like function that recruits Sir1p to telomeres in the mutants, enhancing silencing (see Figure 4B and Table 2). In fact, ectopically expressed Orc1p BAH domain binds more Histone H3 than the Sir3p BAH domain as measured by co-immunoprecipitations, but does not substitute well for the Sir3 BAH domain in silencing [11].

### Docking Sir3p BAH Domain onto the Nucleosome Face

**Figure 7:**
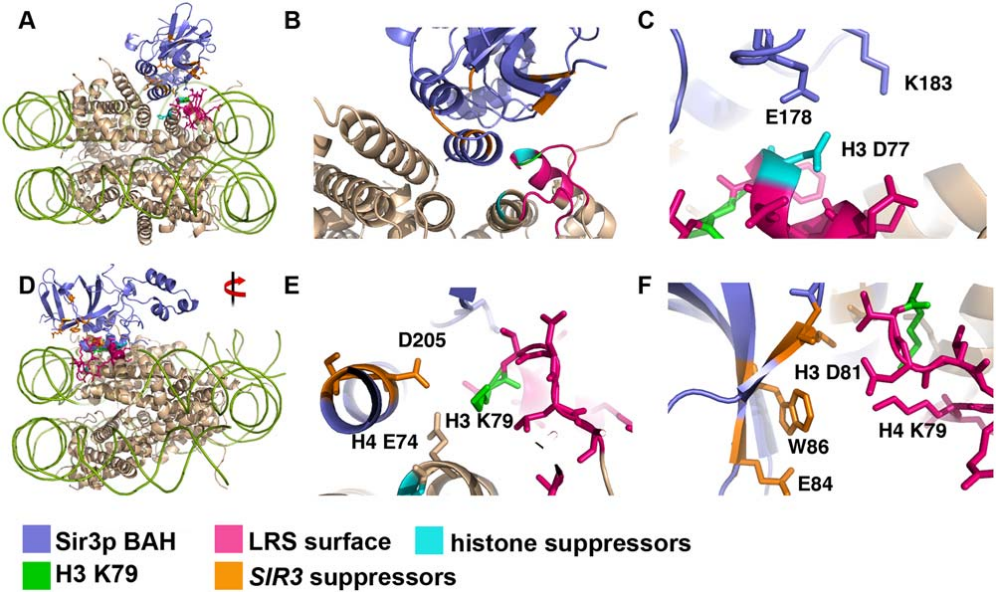
Model for Sir3p BAH domain binding to the LRS surface. The 2Fl7 [35] crystal structure was docked to the 1ID3 nucleosome structure [29]. The helix α8 of Sir3p BAH packs against a group of Histone alpha helices consisting of H2B, H4, and H3. This juxtaposes the LRS surface with the BAH domain found to be important for suppression of LRS alleles. (A) Sighted along the SHL+/−3.5 axis, the DNA was removed to show the details of the docking. (B) A close-up of the docking structure to show the juxtaposition of the LRS surface and the Sir3p BAH domain suppressor residues. (C) An example of potential inhibitory interactions between H3 D77 and Sir3p E178. The introduction of an asparagine at H3 position 77 would ameliorate the inhibitory interactions. (D) A 90° rotation about the X axis from A, showing an overview of the docked structure. The DNA has been removed to show details of the docking. (E) A close-up of the key residues Sir3p D205 and H3 K79. H4 E74 could also be destabilizing, considering its proximity to D205. (F) Showing the interactions between the LRS residues H3 T80, D81, and H4 K79, and key residues W86 and E84. The images were made using PyMOL [62], and the docked structure is available as Datasets S1 and S2.

Genetic suppressors of a defective nucleosome that has lost the ability to bind Sir3p, should reveal side-chains of Sir3 or the nucleosome that would either normally make inhibitory interactions between Sir3 and the nucleosome, or reveal neutral interactions that could be improved by the introduction of a new side-chain. Substitutions of H3 D77 have been isolated as enhancers of telomeric silencing (Table 2). The histone suppressor allele H3 D77N and D77G are the strongest intragenic suppressors of H3 A75V, suppressing both its telomeric and rDNA silencing phenotypes, and the nature of these negative− > uncharged substitutions are consistent with the simple notion that electrostatics underlie LRS surface function. Similarly, many *SIR3* suppressor alleles decrease the negative charge of the BAH domain surface. These results are all consistent with the hypothesis that electronegative regions of the nucleosome and Sir3 normally repel each other in wild-type yeast to some extent and it is the slightly unstable nature of this interaction that underlies silencing. Thus what the mutants do is decrease repulsion, which presumably increases nucleosome binding. Our genetics suggested that two surfaces, one on the nucleosome and one on Sir3p, could directly interact. To this end, we performed studies to attempt to dock the BAH domain on the nucleosome using the mutated “patches” of these surfaces as guides. Considering the large positively charged stretch of amino acids 24 and 34 in *SIR3* we surmised that this section was likely to lie in proximity to the DNA. Additionally, there is a deep cleft in the nucleosome neighboring the LRS domain created by the H2B-H4 four helix bundle at the interface between the H2A/B dimer and the H3/4 tetramer (see Figure 7A and 7B). Guided by these constraints, the helix α8 of the BAH domain was readily docked into this cleft, maintaining the packing of the α-helices of H4 and H2B that make up the proposed docking site (see Figure 7B). The area buried in this structure is approximately 2300 Å^2^, consistent with a strong interaction. Importantly, this configuration directly juxtaposes the LRS domain with the surface of the BAH domain identified here (Figure 7A–7F). We looked deeper into the structure to find evidence of consistent electrostatic interactions between the two proteins. Remarkably, we found that many residues of both surfaces not only line up well, with few clashes, but importantly, we found adjacencies/interactions consistent with the observed silencing-inhibitory and silencing-enhancing mutations isolated (Figure 7). The most frequently isolated *SIR3* suppressor mutants were *D205N*, *L138P*, and *W86R*. The importance of these residues in the docking structure are demonstrated in Figure 7E and 7F. *SIR3 D205* and *L138* are all in close proximity (within 5 Å) to each other and to H3 K79. Given the importance of H3 K79 methylation in Sir3p binding to the nucleosome, we also found in this proposed docking scheme that tri-methylation of H3 K79 is predicted to sterically hinder binding of Sir3p to the LRS surface (Figure 7E). *SIR3 W86* is within 5 Å of H3 T80, H3 D81 and H4 K79. A substitution of an arginine (R) at position W86 could enhance an electrostatic interaction between H3 D81 and Sir3p. Also H3 D77 makes potentially inhibitory repulsive interactions with Sir3p-E178 (Figure 7C), which would be ameliorated by introduction of glycine (G) or asparagine (N) residues into either partner. There are of course stabilizing interactions that would not necessarily be revealed by our mutagenesis but were revealed in the docking study. While our genetic data revealed important side-chains in limiting the interaction between Sir3p and the nucleosome, the docking model showed that there are potential attractive forces as well, namely the α7 helix of the Sir3p BAH domain makes favorable contacts with helices αC and α3 of H2B.

## Discussion

### Models for LRS Function

We considered three models for the function of the LRS domain (i) recognition by the K79 methyltransferase Dot1p (ii) nucleosome-nucleosome interaction, and (iii) trans factor-nucleosome interaction. At the outset of our investigation we had evidence arguing against the first two models. 1) We found that LRS alleles are not intrinsically deficient in H3 K79 methylation as evaluated by immunoblot or *in vitro* Dot1 methylation assays. Although a few alleles are deficient in methylation, most are not. Therefore, recruitment of Dot1p to effect H3 K79 methylation cannot be the sole function of the LRS surface. 2) The H3 R83A allele, unlike its structurally equivalent H4 R45C SIN allele, is competent to form oligonucleosome arrays *in vitro* by sedimentation analysis, arguing against an inter-nucleosomal interaction defect [9].

To genetically probe the function of the LRS surface, we used EMS mutagenesis to find suppressors of H3 A75V loss of telomeric silencing. In this screen almost half of the mutations were in histone H3 and H4 genes. Remarkably, all the remaining chromosomal mutations, which were dominant and therefore difficult to characterize, were subsequently found to map to *SIR3* and to have point mutations in the Sir3p BAH domain that accounted for the suppressor phenotype. This fact alone provides strong evidence consistent with a Sir3p BAH - LRS surface interaction underlying silencing. However, it was formally possible that the Sir3p BAH domain could have an indirect effect on the LRS surface. We have three additional lines of evidence that argue against such an indirect effect. (i) *SIR3* suppressor alleles restore Sir3p binding to telomeric DNA in H3-A75V mutants (ii) the electrostatic alterations in the mutants in H3 that lose silencing, and the H3-A75V suppressing mutants in *SIR3* and *hht2* (H3) that restore that silencing, suggest complementary physical interactions, and (iii) *SIR3* suppressor mutant residues lie in a contiguous surface similar in dimensions to the LRS surface.

To provide evidence for a direct LRS-Sir3p interaction we used ChIP analysis of Sir3p and we did indeed find that the LRS mutant H3-A75V was completely defective in binding Sir3p to telomeric chromatin, and that both Sir3p-D205N and Sir3p-L79I completely restored the binding. Based on targeted mutagenesis of the BAH domain of *SIR3* we were able to further genetically define the surface of Sir3p important for suppression of LRS alleles. We also noted that both the D205N mutation and 69% of the mutants found in the targeted mutagenesis study decreased the negative charge of the Sir3p BAH domain, distinctly complementary to the electrostatic trend seen in the H3 A75V histone H3/H4 suppressors located in the LRS domain, raising the possibility that decreasing charge compatibility would decrease nucleosome/BAH affinity and increasing it would restore binding. To test the notion that such charge complementarity may function between the LRS surface and the BAH domain we tested the allele specificity of the *SIR3* suppressor mutants, and found that *SIR3* mutants were able to generally suppress the loss of telomeric silencing of LRS surface mutations, but were not classically allele-specific. Why weren't we able to isolate residue-specific suppressors? The LRS surface may be important for binding to several different proteins/complexes, and a general electrostatic interaction could allow the surface to bind different factors at different loci. While we provide evidence that Sir3p binds to the LRS surface at telomeres, these *SIR3* alleles do not suppress H3-A75V's loss of rDNA silencing. It will be interesting to determine whether other BAH domain containing proteins such as Orc1p, Rsc1p or Rsc2p can bind the LRS surface at other loci in the yeast genome [39],[40].

### Complementary Surfaces Underlie the Sir3p-Nucleosome Interaction

In this mutational study suppressor mutations were used to inform docking experiments between the Sir3p BAH domain and the LRS surface. The interaction between the LRS surface and the Sir3p BAH domain is determined by charge complementarity and surface complementarity, an improvement in either type of complementarity can suppress defects in either type of complementarity. Based on this docking we did find residue-specific interactions consistent with our genetics, and found that the key residues H3 K79 and H3 D77 could play critical roles in stabilizing the docked structure. Specifically, H3 K79 could interact with Sir3p residues W86, L138, and D205, all of which would be inhibited by tri-methylation of H3 K79; such inhibition is presumably steric, i.e. reducing surface complementarity. Additionally, substitutions of H3 D77 are the strongest suppressors of H3 A75V, suppressing both its telomeric and rDNA silencing phenotypes (Figures 2 and 3). Based on the idea that electrostatics underlie LRS surface function, and based on our docking experiments, we predict that H3 D77 normally makes potentially inhibitory interactions with Sir3p E178 (Figure 7), hence the D77N and D77G substitutions most likely function by increasing Sir3p/nucleosome affinity. H3 D77 is important for inhibitory interactions with Sir3p residues, whereas Sir3p D205 maintains inhibitory interactions with both H3 and H4, all of which could be ameliorated by a decrease in negative charge as seen in all of the substitutions of aspartic acid (D) at position 205 of *SIR3* suppressors (Figure 7). Repulsion of electronegative forces limit the interaction of Sir3p BAH domain and the LRS nucleosomal domains, and hence decreasing them leads to greater affinity. Figure 1 shows an electronegative region abutting the LRS region; mutations that remove any positive charge from the LRS surface lead to loss of silencing. We also predict that Sir3p W86 does not normally make inhibitory interactions but introduction of an (R) Arginine could improve interactions with the nucleosome by creating a salt bridge with H3 D81 (Figure 7F). Not all substitutions in Sir3p BAH domain would improve electrostatic complementarity, in fact many of the suppressor mutants change hydrophobic residues, for example L79I and L138P. We predict that altering these side-chains improves surface complementarity between the BAH domain of Sir3p and the LRS surface. The attractive forces may involve the positively charged H4 N-terminal tail and/or the H2B α-C helix that abuts the α7 Sir3p helix; there are many complementary charge interactions.

### Indirect and Direct Histone Suppressor Alleles

Whereas the majority of the H3 A75V suppressor mutations in histones mapped to the LRS surface, three suppressor mutations, H3 R39K, H4 A15V and H4 H75Y are elsewhere. A previous study that sought H3 and H4 mutations that restored telomeric silencing to a crippled *cac1Δ* mutant isolated the same histone alleles that we isolated here that can suppress the H3 A75V telomeric silencing phenotype. Investigating the function of these alleles, Smith *et al.* [31] demonstrated that H4 A15V decreases the acetylation of H4 K16, which in turn interferes with Sir3p binding [11],[21],[26]. Presumably, decreasing the acetylation state of K16 tips the balance toward Sir3p binding at the expense of Dot1p, and thereby increases silencing. Losing H4 K16 acetylation all together could actually hinder silencing because of a dilution effect on silencing complexes, which explains why a H4 K16R mutation or a *sas2* deletion can lead to loss of telomeric silencing [41]. The suppressor mutations H3 R39K and H4 H75Y are more difficult to reconcile. In a follow up study to the original paper that identified these two mutations Xu *et. al.* [42] demonstrated that both H4 H75Y and H4 R39K increased spreading and H4 H75Y also increased the stability of silent chromatin. Additionally, they predicted that a substitution of tyrosine for histidine could increase the H2B/H4 interaction. Considering the proposed docking of the Sir3 BAH domain onto the nucleosome, an increase in stability between H4 and H2B could enhance Sir3p binding and hence increase silencing. Alternatively, the decreased interaction of H75Y with Asf1, predicted to decrease nucleosome disassembly/assembly thereby increasing telomeric silencing could explain its suppression of H3 A75V [43]. Recently H3 R39, along with the residues N terminal to it, was co-crystallized with histone chaperone RbAp46. This residue may have a similar effect as H75Y, except not on the nucleosomal structure itself. Instead this mutation may alter a histone/chaperone interaction, which is important for disassembly/assembly processes that affect telomeric silencing [44].

### The BAH Domain and the Evolution of Silencing Mechanisms

Homologs of *SIR3* have not been found in other fungal taxa outside of *Saccharomyces*. However, all eukaryotes share an essential replication protein, Orc1p, the N-terminus of which consists of a BAH domain similar to that of Sir3p. This fact suggests that Sir3p's role in silencing was recently acquired in evolution, and study of the two proteins' sequence might give some hints as to functional constraints on their evolution. The similarity in structures suggests that Orc1p's BAH domain might also contact the LRS surface as part of its role in defining origins of replication. Indeed, a recent study of mammalian Orc1p BAH domain suggests a specific role for this domain in chromatin binding, and this domain expressed ectopically is able to co-immunoprecipitate histone H3 in yeast [11],[45]. Other BAH domains are found in proteins important for DNA methylation and transcription, consistent with a generic role in binding the LRS surface [39].

We observe that the majority of the mutations we isolated in *SIR3* that suppress LRS surface defects are “Orc1-like”, i.e. the amino acid substitutions are in equivalently positioned residues that differ between these two proteins and the mutations correspond to the residues found in Orc1p (Figure 4B). Additionally we predict by our modeling that these mutations would increase the affinity of Sir3p BAH domain for the nucleosome and show that in vivo they ChIP more telomeric DNA than wild type Sir3p. Counter intuitively, our suppressor Sir3 alleles do not silence as well with wild-type histones as does wild-type Sir3p. Perhaps, as in the case of the Orc1p BAH domain, binding too strongly to chromatin is not productive for telomeric silencing [11],[12],[45]. Following the presumed gene duplication event that gave rise to *SIR3*, its BAH may have acquired a lower affinity for the LRS surface, consistent with the fact that Sir3p seems to have at least one additional distinct chromatin-binding domain [21],[26]. Further, a role requiring alternating binding and release of chromatin, or allowing for conformational changes that might be required for silencing [11],[46]. Sir3p might require a chromatin binding affinity in a distinct “sweet spot” in between that required to stabilize a silencing competent structure but sufficiently weak to allow the necessary dynamic structural changes.

### Sir3p and the Chromatin Fiber

There are two models of the chromatin fiber, solenoid model and the two-start helix model [10],[47],[48]. In the two start helixmodel the nucleosomes make a zig-zag formation with every other nucleosome facing each other, while in the one-start solenoid model nucelosomes are inter-digitated. Studies of chromatin condensation *in vitro* used purified recombinant nucleosomes lacking the modifications seen *in vivo* and their condensation is highly dependent on salt and histone tail domains [46],[49]. *In vivo*, perhaps other proteins actually mediate nucleosome-nucleosome interactions. *SIR3* could play this role at the telomeres. In the fiber models proposed by Schalch et al. or Robinson et. al. [10],[48], the LRS surface is solvent exposed and available for Sir3p binding. However full engagement of Sir3p would result in a distortion of either structure, and suggests that the actual higher order structure of silent chromatin could be significantly different.

## Materials and Methods

### Strains and Media

All yeast strains are described in Table 3. Yeast media used for silencing assays were as described previously [50], except that 0.8 mM adenine was added to Pb2^+^-containing medium. The drugs clonNAT (Nat) and hygromycin (Hyg) were used as described [51] to select for transformants. G418 was used as previously [52]. Yeast media used for TaGAM were as previously described [53].

**Table 3:** Strains used.

Strain or Plasmid	Genotype and Characteristics	Reference or Source
**Strain**		
JPY12	*MAT* ***a*** * his3200 leu21 lys20 trp163 ura3-167 met150 ade2::hisG RDN1::mURA3/HIS3 RDN1::Ty1-MET15 TELV::ADE2 hht2-hhf2Δ::hygMX hht1-hhf1Δ::natMX pJP11 (LYS2 CEN HHT1-HHF1)*	[1]
ANY34	*MAT* ***a*** * ura3-52 lys2-801 ade2-101 trp163 his3200 leu21 ppr1Δ::HIS3 adh4Δ::URA3-TEL-V11L VR-ADE-TEL hht2-hhf2Δ::hygMX hht1-hhf1Δ::natMX pJP11 (LYS2 CEN HHT1-HHF1)*	[9]
ANY59	*MAT* ***a*** * ade2-101 his3-200 leu2-1 lys2-801 trp1-63 ura3-52 ppr1Δ::LYS2 adh4Δ::URA3-TEL-VIIL TEVR- ADE2 hht1-f1Δ::Nat hht2-f2Δ::hyg-hht2 A75V-HHF2 can1Δ::LEU2-MFA1pr-HIS3*	This study
ANY60	*MATα ade2-101 his3-200 leu2-1 lys2-801 trp1-63 ura3-52 ppr1Δ::HIS3 adh4Δ::URA3-TEL-VIIL TEVR- ADE2 hht1-f1Δ::Nat hht2-f2Δ::hyg pA75V CEN LEU2*	This study
ANY61	*MAT* ***a*** * ade2-101 his3-200 leu2-1 lys2-801 trp1-63 ura3-52 ppr1::HIS3 adh4Δ::URA3-TEL-VIIL TEVR- ADE2 hht1-f1Δ::Nat hht2-f2Δ::hyg pA75V CEN TRP1*	This study
ANY70	*MAT* ***a*** * ade2-101 his3-200 leu2-1 lys2-801 trp1-63 ura3-52 ppr1Δ::HIS3 adh4Δ::URA3-TEL-VIIL TEVR- ADE2 hht1-f1Δ::Nat*	This study
ANY71	*MAT* ***a*** * ura3-52 lys2-801 ade2-101 trp163 his3200 leu21 ppr1Δ::HIS3 sir3Δ::KANMX adh4Δ::URA3-TEL-V11L VR-ADE-TEL hht2-hhf2Δ::hygMX hht1-hhf1Δ::natMX pDM18-A75V (TRP1 CEN hht1-A75V-HHF1) pAN17*	This study
ANY72	*MAT* ***a*** * ura3-52 lys2-801 ade2-101 trp163 his3200 leu21 ppr1Δ::HIS3 sir3Δ::KANMX adh4Δ::URA3-TEL-V11L VR-ADE-TEL hht2-hhf2Δ::hygMX hht1-hhf1Δ::natMX pDM18 R78G (TRP1 CEN HHT2-hhf2-R78G) pAN17*	This study
ANY73	*MATα ade2-101 his3-200 leu2-1 lys2-801 trp1-63 ura3-52 TEVR- ADE2 hht1-f1Δ::Nat hht2-f2Δ::hyg pJP11(HHT1-F1 CEN LYS2)*	This study
**Plasmid**		
pJP11	*LYS2 CEN HHF1-HHT1*	[1]
pJP15	*LEU2 CEN HHT2-HHF2*	Park and Boeke unpublished
PDM18	*TRP1 CEN HHT2-F2*	[63]
pAN17	*LEU2 CEN SIR3*	This study
pAN18	*LEU2 CEN SIR3-D205N*	This study
pAN19	*LEU2 CEN SIR3-L79I*	This study
pAN20	*LEU2 CEN SIR3*	This study
pLP304	*LEU2 2micron SIR3*	[54]

### Plasmids

pAN17 (*SIR3*, *LEU2* CEN) was made by digesting the *SIR3* containing plasmid plP30 [54] with *Sal*I and cloning it into the *Sal*I site of pRS415. pAN18 *(sir3-D205N)* and pAN19 *(sir3-L79I)* were cloned by PCR of *SIR3* from suppressor strains and exploited the endogenous *Apa*I site 530 bp upstream of the *SIR3* ORF and the *Bmg*BI site internal to the gene. After digestion with those enzymes the PCR products were subcloned into a similarly digested pAN17 vector.

### Yeast Strains

The strains used are summarized in Table 3. ANY59 was made by crossing ANY34 with YPH499 and isolating tetrads that were *MAT* ***a*** *ade2-101 his3-200 leu2-1 lys2-801 trp1-63 ura3-52 ppr1::HIS3 adh4::URA3-TEL-VIIL TEVR-ADE2 hht1-f1::Nat*, followed by successive transformations with a *ppr1::LYS2* cassette, integration of *hht1-f1::Nat hht2-f2::hyg-hht2 A75V-HHF2*, and finally *can1Δ::LEU2-MFA1pr-HIS3*. ANY73 was a spore from the cross between ANY34 and YPH499. ANY60 and ANY61 are spores from a cross between ANY34 and ANY73. ANY71 and ANY72 were made by a one-step PCR-transformation method that resulted in the replacement of the *SIR3* open reading frame with the *KANMX4* cassette [51] of ANY34 followed by transforming with their respective *SIR3* containing plasmids.

### PCR Mutagenesis of *SIR3* BAH Domain

We constructed mutated derivatives of the *SIR3 LEU2* plasmid pAN17 by transforming ANY71or ANY72 cells and selecting Trp^+^ Leu^+^ colonies. These were then replica-plated to SC+FOA. The mutations were identified by DNA sequence analysis and confirmed by retransformation. SIR3 BAH domain mutagenesis was done using PCR under the following conditions: 20 ng pAN17, 0.1 mM MnCl_2_, 1.5 mM MgCl_2_, 0.4 M primers, 0.1 mM dNTPs and 0.1 U *Taq* polymerase; 30 PCR cycles of 95°C for 1 min, 55°C for 0.5 min and 72°C for 1 min. A gapped pAN17 derivative was also generated by PCR using the Expand Long template PCR system (Roche Biosciences), using recommended conditions for buffer 2, generating a gap spanning the *SIR3* BAH domain. Approximately 300 ng of mutagenized PCR product and 150 ng of gapped plasmid PCR product were co-transformed into ANY71 or ANY72 cells yielding 20,000 Leu^+^ Trp^+^ cells. Approximately 600 colonies were pink in color and FOA^r^, 300 of which were sequenced by PCR.

### Histone H3 A75V Suppression Screen

*MAT* **a** and *MATα* versions of reporter strain ANY60 and ANY61 were streaked to single colonies and then grown overnight in 7 mL YPD. *MAT* **a** versions carried *TRP1*-marked plasmid pDM18-H3 A75V and *MATα* versions contained *LEU2*-marked plasmid pJP15. 1 mL of each overnight culture was transferred to a microfuge tube, washed twice with water and resuspended in EMS buffer. 30 µL EMS was added to each tube and incubated at room temperature for one hour. Cells were washed with 1 M Na thiosulfate three times and the A_600_ was measured. Approximately 10^7^ cells were plated each onto a total of 100 10 mm YPD plates and incubated overnight at 30°C. Cells were replica-plated to SC+0.1% FOA with 0.8 mM adenine and incubated for 5 days at 30°C, the plates were then replica-plated again to SC+0.1% FOA with minimal adenine, grown for 3 days at 30°C and then transferred to 4°C for 5 days to develop the pink color. Cells that were both pink and FOA^r^ were picked and streaked to single colonies. Single colonies were patched to SC−Leu or SC−Trp medium and replica plated to SC−Ura and SC−Ade to test for retention of the telomeric silencing markers. To isolate plasmid dependent suppressors, cells were transformed with a clean plasmid containing H3 A75V and wild-type H4. The original plasmids were then recovered from suppressor strains that lost their ability to suppress upon retransformation with new H3/H4 plasmid. The remaining suppressor strains were tested for dominance and their mutations were mapped using TaGAM.

### TaGAM (TAG Array Mapping) Procedure

The general strategy used to map the dominant suppressor mutations (all of which were subsequently shown to reside in *SIR3* by PCR amplification, sequencing, and phenotypic reconfirmation in the context of full length *SIR3*) was to cross the suppressor strain, which is 5-Foa resistant due to silencing of the telomeric *URA3* gene, to a bar coded pool of haploid mutant yeast, in which all nonessential ORFs are individually knocked out using a *kanMX* marker. The meiotic recombination frequencies between the suppressor and the barcodes, which span the genome were measured using a microarray readout as in SLAM (Synthetic Lethality analyzed by Microarray; [53]). The recombinant spores were selected for the *URA3* marked telomere (Ura^+^), the mutant histones (Hyg^R^), and the bar coded kanMX alleles (G418^R^). A control pool was made in parallel and differed from the experimental pool only in that it was never selected for 5-Foa resistance.

Individual suppressor strains to be mapped were crossed with strain ANY59, sporulated and then germinated on MM (magic medium) to obtain an integrated form of H3 A75V and the SGA reporter, which allows subsequent selection of *MAT* **a** spores from a sporulated culture [55]. Suppressor-containing spores were selected by growth on 5-Foa, canavanine resistance, His^+^ phenotype and pink color. These *MAT* **a** suppressor derivatives were then crossed with a pool of the *MAT*α haploid knockout collection. Approximately 5×10^5^ log phase cells of both the pool and each individual suppressor strain were mixed well in 1 mL YPD. Cells were plated onto 7 10 mm YPD plates and incubated overnight at 22°C for mating. Cells were harvested from the plates and to obtain diploids; 9×10^5^ of these cells were plated onto 7 100 mm YPD+Nat+G418 (Nat is nourseothricin) plates incubated 2 days at 30°C and replica plated again to YPD+Nat+G418 for 1 day at 30°C. Cells were harvested and aliquots of diploid cells were frozen. 150 OD_600_ of diploids were grown in 300 mL YPD for three hours, washed with water and resuspended in 300 mL sporulation media. Cells were sporulated for 5 days at 25°C and then 5×10^9^ spores were plated onto one 100 mm SC+Can+Nat+Hyg+G418−Leu−His—Arg−Lys−Ura plate, grown for 5 days at 30°C, replica-plated again to the same medium, and incubated at 30°C for 2 days. Finally, the cells were replica plated to SC+5-Foa+Ade for the experiment (selecting for the suppressor) and SC−Ura+Ade (selects for the presence of the URA marked telomere) for the control. gDNA was made from both pools of yeast, PCR of the barcodes of control gDNA was labeled using Cy5 and the experimental gDNA was labeled using Cy3. The PCR reactions were hybridized to the Hopkins TAG array as described previously [53],[56]. Cy5 and Cy3 ratios were normalized to overall intensity in both channels and the data were plotted using Treeview [57] to produce a karyoscope of the Cy5/Cy3 ratios.

### Chromatin Immunoprecipitation (ChIP)

Sir3p binding was analyzed in ANY71 transformed with pRS414, pAN17, pAN18 or pAN19. ChIP was performed as previously described [58] with optimization. For each strain 100 mL cells were grown in SC−Ura medium and grown to an A_600_ of 0.8–1.2, cross-linked with 1% Formaldehyde for one hour, washed 2× with PBS, and frozen as pellets. The equivalent of 15–20 OD's and 2 µL polyclonal Sir3p antibody (a kind gift of Danesh Moazed) per IP were used. IP and input were analyzed using the Applied Biosystems SYBR green RT-PCR system. Each immunoprecipitation was normalized relative to the IP observed for the nonspecific *PHO5* locus and input DNA. The data shown are averages of at least three independent experiments. The primers used were as described previously [9]. The dependence of the ChIP signals on the Sir3p antibody was confirmed by performing mock ChIP experiments in the absence of antibody and performing ChIP experiments with an empty pRS415 vector.

### Immunoblotting

Whole–cell extracts of yeast cells were obtained by NaOH as described previously [59] Samples were denatured in boiling SDS sample buffer, resolved by SDS–PAGE, transferred to nitrocellulose membranes and probed with indicated antibodies; mouse monoclonal anti-αtubulin (Sigma–Aldrich, T5168) rabbit polyclonal anti–Sir3 (a gift from Danesh Maozed).

### rDNA Silencing Assays

Silencing strength in the ribosomal DNA (rDNA) was assessed with the *mURA3/HIS3* reporter by serial dilution on SC−His medium to prevent elimination of the rDNA reporter containing 0.1% 5-Foa to assay down-regulation of rDNA::*mURA3*. Silencing strength of the telomeric DNA was assayed by serial dilution on SC−Ura. Serial dilutions were performed as follows. Cells were scraped from the plates and resuspended in 100 µl of sterile water. The cell suspension was normalized to an *A*_600_ reading of 0.3 and then serially diluted in 5-fold or 10-fold increments; 5 µL of each dilution was spotted onto either nonselective or selective agar plates using a 12-channel pipette. Plates were incubated for 2 to 5 days.

### Colony Color Silencing Assays

rDNA silencing was also assayed using the *MET15* color assay. Strains to be tested were plated onto lead (MLA) plates to give approximately 100 to 200 colonies per plate. The plates were incubated at 30°C for 8 days and then photographed. Telomeric silencing was also assayed using the *ADE2* color assay. Strains to be tested were plated onto SC−Trp plates to give approximately 100–200 colonies per plate. The plates were incubated at 30°C for 3 days and then were incubated at 4°C for 3 days and photographed.

### Modeling of the Sir3p/Nucleosome Complex

Docking of Sir3p in the nucleosome was performed using the molecular modeling program Quanta (Accelerys). The Sir3p structural model (PDB ID 2FL7) was manually docked in the nucleosome (PDB ID 1ID3) in a nucleosome cleft abutting the LRS surface. This cleft, formed by the interface between H3/H4 and H2B/H2A, shows surface complementarity with Sir3p's α8 helix. The Sir3p surface is at an optimal distance from the LRS surface to allow the interaction between the regions in Sir3p and the nucleosome that affect silencing. The orientation of Sir3p BAH domain in the complex was guided by the assumption that the basic region (residues 24–34), which is disordered in the crystal structure, interacts with the negatively charged DNA backbone. Protein-protein contacts were optimized only by modifications of the side chain conformations. Initially, suitable side chain conformations were selected from a library of rotamers and further optimized by energy minimization using CHARMm32 (Accelerys) as the force field. Only the side chain atoms of both proteins were allowed to move during the optimization, with all other atoms constrained in a fixed position. Areas buried for the modeled SIR3/nucleosome complex were calculated using the program NACCESS [60].

## Supporting Information

Dataset S1 Sir3p_model.pdb
(0.12 MB TXT)

Dataset S2 nucleosome_model.pdb
(0.95 MB TXT)
